# Literature Review: Management of Primary Atlantoaxial Osteoarthritis and Associated Occipital Neuralgia

**DOI:** 10.7759/cureus.101102

**Published:** 2026-01-08

**Authors:** Bharat R Dave, Ajay Krishnan, Saurabh S Kulkarni, Shivanand C Mayi, Ravi Ranjan Rai, Mirant B Dave, Mikeson Panthackel, Arjit Vashishtha, Amritesh Singh, Yogenkumar Adodariya

**Affiliations:** 1 Spine Surgery, Stavya Spine Hospital and Research Institute, Ahmedabad, IND; 2 Spine, Bhavnagar Institute of Medical Science, Bhavnagar, IND; 3 Spine, Stavya Spine Hospital and Research Institute, Ahmedabad, IND

**Keywords:** atlantoaxial osteoarthritis, c1–c2 lateral mass arthritis, occipital neuralgia, posterior c1–c2 fusion, radiofrequency ablation, surgical management

## Abstract

Primary atlantoaxial osteoarthritis, or C1-C2 (cervical 1-cervical 2) lateral mass arthritis, is an increasingly recognized but often underdiagnosed source of upper cervical and occipital pain, particularly in older adults. Unlike subaxial cervical spondylosis, which typically causes diffuse neck or upper back pain, this condition usually produces localized pain in the upper neck, occiput, or behind the ear, without frequent neurological symptoms. Clinical presentation is often unilateral, with pain exacerbated by lateral flexion and rotation, and diagnosis relies on radiographic and magnetic resonance imaging (MRI) findings showing joint space narrowing, sclerosis, osteophytes, and marrow edema. Conservative management, including nonsteroidal anti-inflammatory drugs (NSAIDs), soft collars, physiotherapy, and corticosteroid injections, is effective for many patients, but persistent, refractory pain may necessitate surgical intervention such as posterior C1-C2 fusion, which provides robust segmental stabilization and lasting relief. Emerging minimally invasive techniques, such as computed tomography (CT)-guided radiofrequency ablation of the C2 dorsal root ganglion, may offer an intermediate option for select patients. Most studies guiding management are retrospective, underscoring the need for prospective research to refine diagnostic and therapeutic approaches for this condition.

## Introduction and background

Lateral atlantoaxial osteoarthritis (AAOA), also known as C1-C2 (cervical 1-cervical 2) lateral mass arthritis (LMA), is a relatively uncommon yet increasingly recognized source of pain originating in the upper cervical and occipital regions. Unlike secondary C1-C2 arthritis associated with systemic diseases such as rheumatoid arthritis, primary C1-C2 arthritis occurs in the absence of an underlying systemic inflammatory disorder [1]. Despite growing clinical awareness, the current understanding of LMA remains limited, with available evidence derived mainly from retrospective studies. Emerging research underscores the need for improved diagnostic criteria and targeted therapeutic approaches to better address this overlooked source of cervicogenic pain.

AAOA is a rare degenerative condition of the upper cervical spine that affects about 4% of patients in outpatient practice. Most affected individuals are women aged 60-80 years [1]. The lateral joint between the atlas and axis is usually the primary pain origin, often radiating through the greater occipital nerve (GON).

Because of its rarity, this condition is often mistaken for more common disorders such as tension-type headaches or pain arising from subaxial cervical spondylosis. Consequently, prompt and accurate diagnosis is crucial to prevent prolonged suffering and avoid unnecessary delays in effective treatment [2].

Most studies guiding AAOA management use retrospective data. With this review, we focus on the current literature guiding management strategies, identify their limitations, and highlight the future scope of management of AAOA.

## Review

Methodology

Search Strategy

A focused literature review was conducted on primary atlantoaxial (C1-C2) osteoarthritis using the keywords "C1-C2 arthritis", "atlantoaxial joint osteoarthritis", and "primary C1-C2 arthritis", restricted to English-language publications from 2010 to 2025, with prioritization of studies from the past 15 years while allowing inclusion of earlier foundational works when necessary to support key concepts. The search was performed in the MEDLINE database (PubMed), excluding preprints, and the Boolean operator "OR" was used to combine the selected terms for comprehensive retrieval. The data has been screened by 

Eligibility Criteria and Study Selection

Eligible study designs included case reports, meta-analyses, multicenter studies, observational studies, comparative studies, randomized controlled trials, systematic reviews, and narrative reviews. This ensured breadth and depth across evidence hierarchies. Screening yielded 52 results, of which 14 studies met the inclusion criteria for primary AAOA published after 2010. Exclusions removed studies addressing secondary osteoarthritis due to rheumatoid arthritis, psoriatic arthritis, trauma, non-AAOA-relevant topics, publications prior to 2010, and animal studies to maintain clinical relevance to the primary disease. Quantitative pooling was not performed due to clinical and methodological heterogeneity, and a qualitative narrative synthesis was used instead.

Anatomical significance

The distinctive structure of the lateral C1-C2 joint makes it particularly prone to both shear and compressive stress. Approximately 50% cervical spine rotation occurs at this segment. Because the atlas (C1) lacks a vertebral body, its lateral masses are responsible for bearing and transmitting the weight of the head. Usually, the lateral masses of the C2 vertebra are symmetrical, tilting outward by about 20° on the coronal plane and remaining nearly parallel to the horizontal plane on the sagittal view [3,4]. The preserved, symmetrical anatomy of these lateral masses is essential for maintaining the stability and biomechanical equilibrium of the atlantoaxial joint (AAJ).

Pathophysiological background

Few recent studies suggest that an asymmetry in the C1-C2 lateral masses forms the pathological basis for morphological changes in the AAJ. These studies also identified that a unilateral high-riding vertebral artery (HRVA) leads to an asymmetrical settling of the C2 lateral mass. Asymmetry in AAJ leads to uneven biomechanical stress concentration on one lateral mass, thus accelerating degenerative changes in AAJ [5,6]. In addition, the articular surfaces of the C1-C2 zygapophyseal joints are larger in diameter and area compared with the other cervical facets. As much as 85% of the gliding motion is coupled with atlantoaxial rotation. Pain originating from the C1-C2 facet in LMA can be referred, as seen in other arthritic conditions, or may present as radicular pain. To support this, experimental distention of the C1-C2 facet capsule with intra-articular saline has been shown to reproduce occipital and postauricular pain in patients with LMA, and even in individuals without arthritis. The proximity of the C2 dorsal ramus, running directly behind the C1-C2 joint, makes it susceptible to irritation by degenerative osteophytes, potentially leading to radicular symptoms. It is documented that some long-standing, chronic cases can progress to autofusion [7-9].

Some independent risk factors identified leading to AAOA include age, repetitive head-loading occupations (e.g., Indian railway porters), and possible trauma [10].

Some chronic cases can progress towards true spontaneous autofusion (ankylosis) of the C1-C2 joint. Precise prevalence rates for autofusion are not well established, but it is generally considered uncommon, often identified incidentally or in advanced cases [11,12]. 

Clinical features

The more familiar axial discomfort of subaxial cervical spondylosis often presents as diffuse neck or upper back pain, whereas LMA typically produces localized pain in the upper neck, occiput, or behind the ear [1]. It is distinct from both cervical spondylosis and atlantodental joint degeneration because it generally does not cause neurological symptoms such as myelopathy or radicular pain in the arms [1]. As documented by Dave et al. [13], patients present with unilateral upper neck or occipital pain radiating behind the ear, often sharp. Pain is often exacerbated by provocative movements such as lateral flexion and head rotation toward the affected side. Axial loading may further trigger the pain, and traction relieves it. Typical examination findings include reduced cervical range of motion, tenderness over the C1-C2 joint, and occasional crepitus. Some also describe visual strain and grating sensations during neck motion [13].

Radiologic evaluation and diagnosis

AAOA is typically identified on an open-mouth odontoid (OMO) radiograph. Radiographs and CT scans characteristically show unilateral arthritis with joint space narrowing, sclerosis, osteophyte formation, and cystic degeneration. A significant reduction in the atlantoaxial facet angle was observed on the affected side, correlating with symptom severity [13]. On MRI, areas of active inflammation show marrow edema, which is most clearly observed on short tau inversion recovery (STIR) or fat-saturated T2-weighted sequences. These imaging techniques suppress the fat signal, enhancing the visualization of water content, which appears hyperintense. Additionally, fat-saturated post-contrast T1-weighted images effectively demonstrate inflammatory changes within the bone, joint, and adjacent soft tissues [13]. CT and MRI confirm joint degeneration and exclude alternative causes such as rheumatoid arthritis, tumors, or infections.

Primary degenerative osteoarthritis of the AAJ may lead to craniovertebral junctional instability, a periodontoid soft-tissue mass, and basilar invagination, as documented in a retrospective analysis of 108 patients by Goel et al. [14]. All patients exhibited mobile atlantoaxial dislocation. The dislocation was classified as “subtle” when the change in the atlantodental interval between head flexion and extension was 3 mm or less. In contrast, a variation greater than 3 mm was defined as “hypermobility.”

CT angiography (CTA), magnetic resonance angiography (MRA), and digital subtraction angiography (DSA) are rarely used in the diagnosis of C1-C2 (atlantoaxial) arthritis, as their primary role is to assess vascular anatomy or complications rather than joint pathology. These imaging modalities are considered in preoperative planning of surgical candidates or in exceptional cases, such as when vertebral artery compression or anomalous course is suspected secondary to inflammatory or degenerative changes at the AAJ, as documented by Ikedo et al. [15] in a case report of extracranial internal carotid artery dissection caused by compression from a giant osteophyte due to AAOA.

Diagnostic injections can be utilized and are generally of two types: C1-C2 facet capsule distention with saline, which can reproduce the characteristic pain, and anesthetic facet block, which may provide pain relief [10]. Following a change in the sentence, a revised reference supporting the same has been made - bone scans rarely have a diagnostic role in primary AAOS, with its application limited to monitoring disease activity in AAOS secondary to rheumatoid arthritis [11].

Conservative management

Conservative treatment remains the cornerstone of initial management, particularly for mild-to-moderate or early-stage disease.

Initial management includes non-invasive methods like nonsteroidal anti-inflammatory drugs (NSAIDs), soft cervical collars, and physical therapy. NSAIDs form the first line of management for pain and inflammation control. Paracetamol or a weak opioid may be added if NSAIDs are insufficient. Muscle relaxants can be added for associated muscular spasm. There was a significant improvement in Visual Analog Scale (VAS) and Neck Disability Index (NDI) scores at two-year follow-up in patients receiving conservative management [16]. Use of orthotic supports, such as a soft cervical collar, was recommended around the clock to achieve adequate immobilization and pain control. Occurrence of autofusion eliminates the need for any further intervention; however, in symptomatic cases, if these approaches are inadequate, fluoroscopy-assisted joint injections using corticosteroids and local anesthetics can be applied [11,12]. Those patients who did not respond to medical management were offered a GON block, with a maximum of three injections at six-week intervals. Persistent non-responders with unbearable pain were subjected to surgical management with atlantoaxial fusion [16]. Image-guided intra-articular corticosteroid injections can provide over 50% temporary pain relief for up to three months [16-18]. In addition to systemic adverse effects of corticosteroids, intra-articular injections may also cause complications such as infection, injury to the C2 nerve root, and damage to the vertebral artery. A rare but serious event that has been documented is intradural injection, which can result in quadriparesis and respiratory paralysis [16].

Few studies document the use of CT-guided pulsed or thermal radiofrequency ablation (RFA) of the C2 dorsal root ganglion (DRG) for refractory cases [19-21]. Chazen et al. [19] suggested the indications for this procedure as: occipital neuralgia for >6 weeks, ipsilateral arthrosis of the lateral C1-C2 facet joint identified on CT or MR imaging with accompanying C1-C2 foraminal stenosis, and lack of response to conservative therapy, including medication or occipital nerve blocks. However, the procedure is contraindicated in patients with coagulopathy, defined as an INR greater than 1.5 or platelets less than 50,000/cmm. Additional contraindications include a vertebral artery variant crossing at C1-C2 along the needle's path and a history of contrast allergy. In conclusion, all studies reported that C2 DRG ablation is a safe and effective procedure; however, large-scale trials are needed to establish consistent results.

Surgical management

Indication and Patient Selection

Surgical intervention for AAOA is deemed appropriate in patients with persistent, refractory occipital or upper cervical pain after at least four to six months of exhaustive conservative management (e.g., medication, physiotherapy, occipital nerve block, intra-articular injections). Common radiological and clinical findings include C1-C2 osteoarthritis with lateral mass involvement, persistent pain, or neurological compromise such as myelopathy or progressive deficits. Most patients treated surgically are elderly, often with significant disability and imaging-confirmed instability or degenerative change.

Preoperative Evaluation

Work-up emphasizes the following: detailed pain mapping (unilateral suboccipital/occipital neuralgia, rotation-provoked pain), screening for myelopathy, and excluding RA, trauma, infection, or tumor; CT is used to assess joint space narrowing, osteophytes, lateral mass height, and C2 pedicle/isthmus anatomy, while MRI helps assess marrow edema, C2 root compression, and retro-odontoid soft tissue. The configuration of the vertebral artery and the C2 isthmus (including high-riding vertebral arteries and narrow pedicles) determines whether transarticular screws (TAS), Harms-type C1-C2 fixation, C2 laminar screws, or a hybrid construct is selected.

Posterior C1-C2 Fusion Techniques

Posterior C1-C2 arthrodesis is considered the gold‑standard operation for refractory AAOA-related pain, providing robust segmental stabilization and indirect decompression of the C2 root. It eliminates joint micromotion and neural irritation. Modern constructs include C1-C2 TAS fixation with iliac crest bone graft as described by Magerl and Seeman [22], C1 lateral mass-C2 pedicle screw-rod constructs (SRC) (Goel and Harms technique) [23], and hybrid constructs combining TAS on one side with C1-C2 screw-rod fixation on the other when vertebral artery anatomy or bony constraints preclude bilateral TAS [13]. Modern techniques, TAS and SRC fixation, are favored over wiring techniques because of their superior fixation strength, high fusion rates, and low risk of neurologic injury, which may help avoid issues like hyperlordosis resulting from tensioning of the wiring [10].

In a retrospective series of elderly patients with recalcitrant AAOA, most treated with bilateral C1-C2 SRC and a few with TAS or hybrid constructs achieved fusion in all cases with no perioperative or postoperative complications, along with marked pain and disability improvement on the Numeric Pain Rating Scale and NDI, and the vast majority attained substantial clinical benefit [24]. A larger image-guided series of 23 patients treated with TAS, Harms, or hybrid fixation showed similarly favorable results, with VAS scores improving from about 9.4 to 2.9, NDI from roughly 72% to 19%, and a radiographic fusion rate of 95.5% [25]. In a recent meta-analysis, C1-C2 screw rod fixation compared to C1-C2 transarticular fixation was found to have a slightly higher rate of arthrodesis, a lower risk of screw malposition, and a lower risk of vertebral artery injury [26].

There is an ongoing debate over whether to preserve or transect the C2 nerve root during the surgical management of AAOA. Some studies suggest that C2 root transection can provide superior relief of pain attributed to persistent irritation of the nerve by the degenerated C1-C2 joint. C2 neurectomy may be unnecessary when stable screw fixation is achieved without mechanical root compression or when adequate decompression is performed; however, reported adverse effects of C2 root sacrifice have been limited to scalp hypoesthesia, with no significant functional consequences described [27,28].

Limitations

Most available studies are retrospective with small cohorts, limiting generalizability. Few randomized controlled trials exist. Outcome metrics differ across studies, and potential selection bias arises from the exclusion of nonsurgical candidates. Future research should focus on prospective comparisons between RFA, decompression, and arthrodesis.

## Conclusions

Lateral AAOA is an underdiagnosed source of occipital pain in older adults. Conservative management is the primary mainstay and effective for many, but posterior C1-C2 fusion remains the gold standard for severe, refractory cases, providing reliable and lasting relief. Emerging minimally invasive interventions such as CT-guided RFA may bridge the gap between conservative and surgical approaches.
